# Mitochondrial Dynamics Imbalance: A Strategy for Promoting Viral Infection

**DOI:** 10.3389/fmicb.2020.01992

**Published:** 2020-08-21

**Authors:** Zhihua Ren, Xiaojie Zhang, Ting Ding, Zhijun Zhong, Hui Hu, Zhiwen Xu, Junliang Deng

**Affiliations:** ^1^Key Laboratory of Animal Disease and Human Health of Sichuan Province, College of Veterinary Medicine, Sichuan Agricultural University, Chengdu, China; ^2^The College of Animal Science and Veterinary Medicine, Henan Agricultural University, Zhengzhou, China

**Keywords:** virus infection, mitochondrial fission and fusion, apoptosis, mitophagy, RIG-I-like receptors pathway

## Abstract

Mitochondria are highly dynamic organelles that maintain the dynamic balance of split-fusion *via* kinetic proteins. This maintains the stability of their morphological functions. This dynamic balance is highly susceptible to various stress environments, including viral infection. After viral infection, the dynamic balance of the host cell mitochondria is disturbed, affecting the processes of energy generation, metabolism, and innate immunity. This creates an intracellular environment that is conducive to viral proliferation and begins the process of its own infection and causes further damage to the body. Herein, we discuss the mechanism of the virus-induced mitochondrial dynamics imbalance and its subsequent effects on the body, which will help to improve our understanding of the relationship between mitochondrial dynamics and viral infection and its importance.

## Introduction

A virus is a microbe that parasitizes on cells. It uses the host cell’s metabolic system to synthesize nucleic acids and proteins. By regulating the metabolism and physiological processes of the cell, a virus can change the structure and function of the cells to maintain a living environment that is conducive to virus proliferation (Xia et al., 2014). Mitochondria are important organelles in cells, which produce ATP through oxidative phosphorylation and provide energy for metabolism of various substances in cells. Additionally, they participate in cell processes such as differentiation, metabolism, apoptosis, signal transduction, and natural immunity (Trifunovic and Bratic, 2010; Zhang et al., 2018). Mitochondria are in a dynamic balance of high division and fusion in cells. Through the synergistic effect of several dynamic related proteins, the number, morphology, and overall connectivity of mitochondria are adjusted to meet the needs of cells in different environments. This dynamic balance is mitochondrial dynamics (Mireille and Slack, 2018). Mitochondrial dynamics have an important physiological significance. The occurrence of many diseases, such as inflammation, metabolic syndrome, cardiac dysfunction, neurodegenerative diseases, and cancer, are closely related to the abnormal mitochondria dynamics, and mitochondrial dynamic balance is very sensitive to changes in cell environment and vulnerable to various cell stress states, thus becoming the target of destruction by many viruses. Studies have shown that viruses directly or indirectly target mitochondrial kinetic proteins or change the intracellular environment to disrupt the balance of mitochondrial kinetics, resulting in mitochondrial dysfunction and thereby promoting self-infection and subsequent body damage.

Currently, the mechanisms of mitochondrial dynamics and viral interactions are in the preliminary research stage, and there are many questions to be answered. In this review, we summarized the latest research results on viral infections and mitochondrial dynamics and discussed in depth how viruses affect mitochondrial dynamics and the pathogenesis of the body after dynamics imbalance, which will provide new ideas for future antiviral treatment strategies.

## Overview of Mitochondrial Dynamics

Mitochondria are highly dynamic in the cell, which maintains the dynamic balance of the mitochondrial network through continuous fission and fusion. This dynamic balance is regulated by a variety of dynamic related proteins, and it changes with the needs of cell life activities or external stimulation.

### Mitochondrial Fission

In mammals, mitochondrial fission is mainly mediated by dynamin-like protein 1 (Drp1), which belongs to the GTP enzyme system that is generally located in the cytoplasm. After being activated, Drp1 shifts to the outer mitochondrial membrane (OMM) and forms a polymer, which compresses the mitochondria until they split. Drp1 requires the participation of a variety of accessory proteins to perform its function, including mitochondrial fission protein 1 (Fis1), mitochondrial fission factor (Mff), and mitochondrial dynamics proteins of 49 and 51 kDa (MiD49 and MiD51), which act as ligands for Drp1 on the mitochondria and thereby mediate Drp1 recruitment into the mitochondria (Viviane et al., 2015). In addition to the above accessory proteins, mitochondrial fission is closely related to the endoplasmic reticulum (ER; Ji et al., 2017). Under physiological conditions, ER tubules form membrane contact sites (MCSs) around mitochondria (Wu et al., 2018) and then compress mitochondria in the MCSs to form cleavage sites (Friedman et al., 2011). The ER compression force requires the participation of INF2, which induces the cytoskeleton protein, actin, to gather at the cleavage sites and provide compression power (Korobova et al., 2013). Subsequently, another cytoskeletal protein, myosin II, recruits Drp1 and locates it at the cleavage site (Korobova et al., 2014), where it forms a helical structure and compresses mitochondria to divide (Bui and Shaw, 2013). In addition, the aggregation of Drp1 oligomers and Mff was also observed on the endoplasmic reticulum. Further studies found that these Drp1 could move along the endoplasmic reticulum tubules and were recruited to mitochondria through MCSs to participate in mitochondrial division, while the ER-related Mff can stimulate mitochondrial fission (Ji et al., 2017).

Dynamin-like protein 1 is the key protein in mitochondrial dynamics regulation, and it is targeted by many viruses to disturb the dynamics. In a physiological environment, Drp1 is mostly located in the cytoplasm, and only 3% of Drp1 plays a role in the mitochondria; under mitochondrial fission or stress conditions, more Drp1 is recruited to mitochondria (Losón et al., 2013). The translocation and function of Drp1 depend on the regulation of post-translational modification and the most common modification is phosphorylation. There are two important phosphorylation sites in Drp1, ser616, and ser637 (amino acid numbering corresponds to human Drp1), and it is also a target for many viruses to regulate mitochondrial fission activity (Keck et al., 2017; Krishnan et al., 2018). Generally, Drp1 phosphorylation at S616 promotes its translocation into mitochondria, while phosphorylation at S637 keeps it in the cytoplasm (Naoko et al., 2007). For example, cyclin-dependent kinase 1 (CDK1/ cyclin B), extracellular regulated protein kinase2 (Erk 2) and Ca^2+^/calmodulin-dependent kinase II (CaMKII) phosphorylate Ser616 residues of Drp1, thus enhancing Drp1 migration to mitochondria and fission (Naoko et al., 2007; Kashatus et al., 2015; Shangcheng et al., 2016; Toshiro et al., 2019). The phosphorylation of protein kinase A (PKA) at Ser637 inhibited the GTPase activity of its Drp1 (Chuang-Rung and Craig, 2007); however, phosphorylation of S616 residues, which is induced by CDK5, was shown to inhibit the Drp1 aggregation and mitochondrion fission (Cho et al., 2014). Therefore, more studies are needed to clarify the effect of regulation of phosphorylation by different kinases on Drp1. In addition, the activity of Drp1 is also regulated by ubiquitination, S-nitrosylation, and SUMO, which are well summarized in Khan’s papers (Khan et al., 2015). Fis1 is a C-tail anchored protein, which is evenly distributed on the OMM. Fis1 is the only Dnm1 receptor in yeast cells (Drp1 homolog in mammals), but whether Drp1 can be recruited in mammalian cells remains controversial. Oliver et al. found that deletion of Fis1 inhibited the mitochondrial recruitment of Drp1 and the mitochondrial network extended to a certain extent (Losón et al., 2013), while Lee et al. (2004) found that deletion of Fis1 had no effect on the distribution of Drp1 and there was no significant change in mitochondrial length. A recent study found that human Fis1 can mediate mitochondrial division in the absence of Drp1 (Yu et al., 2019). These results may indicate that Fis1 also plays different roles in different cells, so further research on Fis1 is needed. Mff is also a C-tail anchored protein on the OMM, which is the most important Drp1 receptor. Mff overexpression stimulated Drp1 recruitment, while Mff silencing resulted in enhanced mitochondrial fusion (Otera et al., 2010). When Fis1 and Mff are both deleted, mitochondrial division is mediated by MiDs, and MiDs have a higher affinity for S637 phosphorylated Drp1. Therefore, when MiDs are overexpressed, inactive S637 phosphorylated Drp1 is recruited into mitochondria in large quantities, eventually leading to mitochondrial elongation (Palmer et al., 2011; Zhao et al., 2011). Some of the kinetic related proteins mentioned above, such as Drp1, Fis1, and Mff, are also distributed on peroxisome in addition to mitochondrial localization (Schrader et al., 2016). Peroxisome is also a dynamic organelle, which can produce progeny peroxisome through fission. Its division process is very similar to mitochondria, and Drp1 is the main driving protein, which mediates the fission of peroxisome, while Fis1 and Mff are the linker proteins, which collect Drp1 on the membrane of peroxisome and promote the fission process together (Kim et al., 2016; Fonseca et al., 2019).

### Mitochondrial Fusion

Mitochondrial fusion is a multi-step process, including outer membrane fusion and inner membrane fusion, which involves different fusion proteins. Mitofusin1 (Mfn1) and Mfn2 are GTP enzyme proteins that are located on the OMM, which coordinate to mediate OMM fusion (Santel and Fuller, 2001). Mfns contain heptad repeat regions (HR2), and Mfn1 and Mfn2 on adjacent mitochondria can form homologous or heterologous dimers through oligomerization of the HR2 structures, thus binding the two mitochondria together to complete fusion of the outer membranes (Koshiba et al., 2004). Deletion or mutation of either of the two proteins can lead to the abnormality of mitochondrial morphology. Mfn2 activity is regulated by c-Jun N-terminal kinase (JNK), which phosphorylates Mfn2 after activation, and then, the E3 ubiquitin ligase Huwe1 recognizes phosphorylated Mfn2 and ubiquitinates it for degradation by the proteasome system (Leboucher et al., 2012). Similarly, another E3 ubiquitin ligase, Parkin, can induce ubiquitination and degradation of Mfns and inhibit the fusion of mitochondrial outer membrane when it is recruited into the mitochondria (Su et al., 2014).

Fusion of the inner mitochondrial membrane (IMM) is regulated by optic atrophy 1 (OPA1). OPA1 exists in two isoforms: a long isoform L-OPA1 that is combined with IMM and a short isoform S-OPA1 that is dissociated in the inter membrane space (IMS) where S-OPA1 is formed by shear of L-OPA1 (Song et al., 2007). L-OPA1 achieves selective fusion of mitochondria through the heterotypic action of its GTPase domain with adjacent mitochondrial membrane cardiolipid (CL). Although S-OPA1 can promote this heterotypic fusion, it cannot mediate fusion alone (Nakamura et al., 2010). The IMM protein prohibitin 2 (PHB2) has been shown to stabilize L-OPA1, and its deletion will lead to the loss of L-OPA1 selectivity (Merkwirth et al., 2012). There are two cleavage sites S1 and S2 on L-OPA1, which can produce S-OPA1 through the cleavage of several cleavage enzymes (Song et al., 2007), and cooperate to ensure the quantity balance of long and short subtypes of OPA1. Griparic et al. (2007) found that AAA protease YME1 can selectively shear L-OPA1 at S2 site, and knocking out YME1 will lead to an increase in the overall connectivity of mitochondria. Another matrix AAA (m-AAA) protease can promote the stability of L-OPA1. When m-AAA protease is deleted, it will activate metalloproteinase OMA1 and induce it to shear L-OPA1 at S1 site, which will lead to mitochondrial fission (Ehses et al., 2009). Other stimuli in cells, such as oxidative stress, heat stress, and membrane potential dissipation, can also induce the shearing action of OMA1 (Baker et al., 2014). The processing of OMA1 is also regulated by YME1 and PHB2. After the deletion of these two proteins, the precursor OMA1 (unactivated OMA1) accumulates, which reduces the shearing of L-OPA1 (Anderson et al., 2020).

### Physiological Role of Mitochondrial Dynamics

Mitochondria are energy factories in cells, which provide ATP for cell survival through oxidative phosphorylation. This process is closely related to respiratory chain complex encoded by mitochondrial DNA (mtDNA). Mitochondrial fusion effectively maintains mtDNA content and enhances mitochondrial respiration and ATP generation by promoting the exchange and mixing of mitochondrial contents and complementing damaged mitochondrial alleles (Sato et al., 2006). Although mitochondrial fission is not directly related to bioenergy, it can release the damaged mitochondria from the network and maintain the stability of the network quality. In addition, after knocking out Drp1, the activity of respiratory chain complex and the copy number of mtDNA decrease (Parone et al., 2008). Therefore, inhibiting mitochondrial fission or fusion is not conducive to the regulation of mitochondrial bioenergy. When cells are exposed to certain stress environments, mitochondria will change the balance of fission and fusion and then regulate energy generation in response to changes in the intracellular environment, thus helping cells overcome transient and reversible damage caused by stress (Tondera et al., 2009; Steiner et al., 2018). After Tondera et al. stimulated cells with ultraviolet rays or antibiotics, they found that IMM protein stomatin-like protein 2 (SLP-2) inhibited the shearing of L-OPA1, which led to the formation of highly interconnected networks of mitochondria. This over-fusion promoted the oxidative phosphorylation of mitochondria, significantly increased the production of ATP, and had a certain protective effect on cells (Tondera et al., 2009). Similarly, in the cell model of KCl and NaCl induced ion stress, the fusion of mitochondria and the enhancement of respiration were also observed (Steiner et al., 2018). However, under cold stress, the phosphorylation of Drp1S616 was activated, and mitochondrial fission increased, which led to oxidative phosphorylation decoupling, and finally accelerated the generation of energy to meet the needs of the body (Zhao and Gao, 2019).

Mitochondria are involved in many life activities in cells and have important physiological significance. By changing the shape, quantity, and distribution of mitochondria, division and fusion can ensure the stability of mitochondrial function and the normal development of various life activities and maintain the steady state of cells. Mitochondrial dynamics can not only affect bioenergy production but also have physiological functions of regulating apoptosis and mitochondrial antiviral signaling (MAVS) pathway. In the following, we will make a further review combining with the influence of viral infection.

## Mitochondrial Autophagy

**Figure 1:**
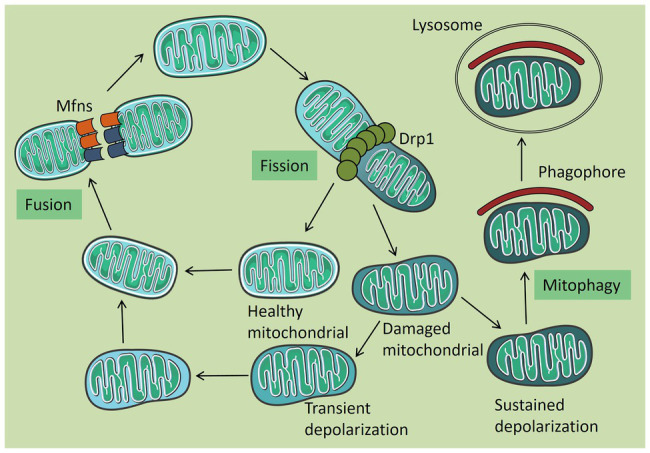
Mitochondrial quality control. Under normal circumstances, mitochondria are tubular in shape and are damaged when subjected to certain pressures. Damaged mitochondria can be released from a healthy mitochondrial network by splitting. Healthy mitochondria return to the mitochondrial network through fusion, and some mitochondria which are temporarily depolarized or not seriously damaged are also fused into the mitochondrial network after repair. The remaining permanent depolarized and irreversibly damaged mitochondria were removed by autophagy.

**Figure 2:**
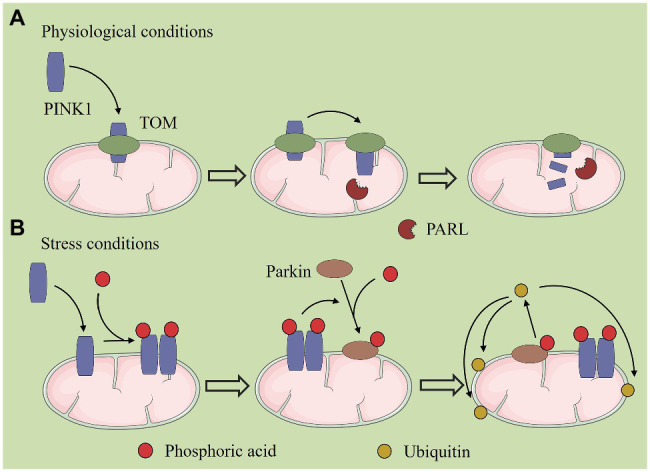
PTEN-induced protein kinase 1 (PINK1)/Parkin-mediated mitochondrial autophagy. **(A)** Under normal conditions, PINK1 is transferred to the inner mitochondrial membrane (IMM) by mitochondrial surface protein transporters, and it is degraded by presenilin-associated rhomboid like (PARL) protein. **(B)** Under stress conditions, PINK1 accumulates in the outer mitochondrial membrane (OMM) and recruits Parkin to phosphorylate it, after which Parkin ubiquitinates a variety of proteins on the mitochondrial surface. Autophagy then recognizes and encapsulates the ubiquitinated mitochondria.

Mitochondrial dynamics and autophagy are two important events that maintain mitochondrial homeostasis, which are closely linked, and they interact with each other and cooperate to complete mitochondrial quality control (Figure 1). Mitophagy is a highly conserved multi-step process that selectively removes damaged mitochondria in cells to maintain the homeostasis of the intracellular environment. In this process, damaged mitochondria are specifically encapsulated by autophagy and then transported to lysosomes for degradation. Mitophagy has two different regulatory mechanisms (Geisler et al., 2010; Novak et al., 2010). The first is mitophagy that is mediated by the two key proteins, PTEN-induced protein kinase 1 (PINK1) and the E3 ubiquitin ligase, Parkin. Under normal conditions, PINK1 is transferred to the IMM by mitochondrial surface protein transporters, and it is degraded by presenilin-associated rhomboid like protein (PARL). Under stress conditions, PINK1 accumulates in the OMM and recruits Parkin to phosphorylate it, after which Parkin ubiquitinates a variety of proteins on the mitochondrial surface. Autophagy then recognizes and encapsulates the ubiquitinated mitochondria (Geisler et al., 2010; Figure 2). In recent years, it has been found that PHB2 can inhibit the activity of PARL, prevent the degradation of PINK1, and promote Thane mitochondrial recruitment of Parkin (Yan et al., 2020). The second mechanism is mediated by receptors that anchor onto the OMM and recruit and interact with the key autophagy protein, LC3, thereby triggering mitophagy (Novak et al., 2010).

During cell growth, the accumulation of reactive oxygen species (ROS), a byproduct of respiration, causes mutation and damage to mtDNA and eventually damages the mitochondrial function. Mitochondrial fission can release these damaged mitochondria from the mitochondrial network and quickly clear them through autophagy (van der Bliek et al., 2013). In addition to elimination by mitophagy, some of the transiently depolarized or not seriously damaged mitochondria can be integrated into the mitochondrial network for repair through mitochondrial fusion. Fusion complements the complete alleles of the two damaged mitochondria to maintain mitochondrial function and the demand for cell biological energy, which is also a key step in the fight against cell aging (Sato et al., 2006; Twig and Shirihai, 2011). Studies have found that before mitochondrion fission, Drp1 interacts with mitochondrial zinc transporter ZIP1 and mediates Zn^2+^ to enter mitochondrial inter-membrane space through coupling mitochondrial calcium uniporter (MCU), thus regulating MMP (Min et al., 2019). However, most Parkin-dependent mitochondrial autophagy is related to MMP loss (Burman et al., 2017). Mitochondrial autophagy is inhibited after interfering with ZIP1 expression. Therefore, the interaction of Drp1-ZIP1 plays an important role in mitochondrial quality control. Some viruses were shown to induce mitophagy by enhancing mitochondrial fission and further inhibiting cell apoptosis to achieve persistent infection. In the following sections, we elaborate on the mechanism of virus-induced mitophagy.

## Viral Infection and Mitochondrial Dynamics

Previous studies have shown that mitochondrial dynamics have an important biological significance, including adapting to the needs of cell metabolism, maintaining cell function, and regulating cell differentiation, and it plays an important role in the body’s resistance to viral infection by participating in innate immune regulation and regulating cell apoptosis. Viruses have developed a variety of strategies to interfere with the dynamic balance of the mitochondria throughout evolution and promote their own infection and proliferation. In this section, we review the mechanisms of different viruses that regulate mitochondrial dynamics and their subsequent effects on the body. These mechanisms are summarized in Table 1.

**Table 1:** The effect of virus on mitochondrial dynamics and its regulatory mechanism, and its subsequent effect on the body.

Virus	Experimental cells	Regulatory mechanism	Result of regulation	Subsequent impact	References
HCV	The human hepatoma cell line (Huh7)	Enhances phosphorylation of Drp1 at S616; Up-regulates Mff expression	Promotes mitochondrial fission	Inhibits apoptosis	Kim et al. (2014)
RV	The monkey kidney cell line (MA104), Human embryonic kidney cell line (HEK293)	Enhances phosphorylation of Drp1 at S616	Promotes mitochondrial fragmentation	Promotes apoptosis and releases virus particles	Arpita et al. (2018)
CMV	HEK293 cells, Hela cells, Human foreskin fibroblasts (ATCC)	Up-regulates the expression of Drp1	Promotes mitochondrial fragmentation	Inhibits MAVS-STING interaction and downstream signal of MAVS	Castanier et al. (2010)
DENV	Huh7 cells; Vero cells	Inhibits phosphorylation of Drp1 at S616	Promotes mitochondrial elongation	Inhibits IFN response, promotes virus replication	Chatel-Chaix et al. (2016)
HIV	Rat Cortical Neurons	Down-regulates the expression of Fis1, inhibits phosphorylation of Drp1 at S637	Disturbs mitochondrial dynamic balance and mitochondrial movement	Induces neuron damage and death	Avdoshina et al. (2016); Rozzi et al. (2018)
PRV	Embryonic rat SCG neurons	Increases cytoplasmic Ca^2+^ concentration, activates GTPase Miro	Promotes mitochondrial fission and disrupts mitochondrial transport	Induces neurons damage and promotes virus growth and spread	Kramer and Enquist (2012)
EBV	Human breast cancer cell line (MCF7)	Activates Notch pathway and up-regulates the expression of Drp1	Promotes mitochondrial fission	Enhances cell migration and tumor cell spread	Deb et al. (2014)
NDV	Chicken fibroblast cell line (DF-1), A549 cells	Up-regulates the expression of Drp1, Mff, Mfns, etc	Disturbs the distribution and morphology of mitochondria	Promotes cell syncytium formation and induces CPE	Ren et al. (2019)
CBV	Mouse embryonic fibroblasts (ATG5KO), Mouse skeletal myoblasts (C2C12), Hela cells	Increases activity of drp1	Promotes mitochondrial fission	Induces cells release EMVs, promotes virus spread	Jon et al. (2017)

### Viruses Disturb Mitochondrial Dynamics to Affect Cell Apoptosis

Apoptosis is an active death process under physiological or pathological conditions, and it is strictly regulated by a variety of genes to maintain the homeostasis of the internal environment. It can be caused by a variety of stimuli. Based on the different initiation stages, apoptosis can be divided into three pathways. In this article, we mainly discuss the pathway that is closely related to mitochondria, i.e., the cytochrome c (Cyt C)-mediated apoptosis pathway. When cells are induced to undergo apoptosis, pro-apoptotic Bcl-2 family members (Bax and Bak) form an oligomer complex that is inserted into the OMM, triggering mitochondrial outer membrane permeability (MOMP), accompanied by the release of apoptotic factors such as CytC from the mitochondria. This Cyt C binds to apoptosis protease activating factor 1 (APAF1) to form an apoptosis complex that activates the caspase-9 precursor, which then cleaves caspase-3 and caspase-7 to activate them. These two proteases selectively degrade proteins in cells, thereby inducing apoptosis (Xiong et al., 2014). The significance of apoptosis for viral infection was shown to be related to the proliferation cycle of the virus. When apoptosis occurs in the early stage of virus replication, proliferation of the virus is inhibited, whereas when apoptosis occurs after the virus matures, the spread of the virus is promoted, cell function loss occurs, and a series of secondary pathological changes that lead to clinical symptoms takes place. Therefore, there are two main ways for viruses to regulate cell apoptosis: on the one hand, viruses can inhibit apoptosis to achieve their own purpose of the persistent infection; on the other hand, viruses also stimulate and induce cell apoptosis (Faletti et al., 2015; Kennedy, 2015).

In the early stage of viral infection, cells block virus replication and transmission through apoptosis, which is a cellular defense mechanism (Xia et al., 2014). Therefore, many viruses have evolved strategies to inhibit apoptosis, for example, vMIA, a viral protein of cytomegalovirus (CMV), inhibits the permeability of mitochondrial outer membrane by recruiting Bax into mitochondria and binding with it, thus blocking Bax-mediated apoptosis (Arnoult et al., 2004). Here, we mainly discuss viruses that inhibit apoptosis by regulating mitochondrial dynamic. Hepatitis C virus (HCV) is a common RNA virus, which usually causes chronic hepatitis after infection. In HCV-infected cells, apoptosis was significantly inhibited (Kim et al., 2014), which was closely related to the increase of mitochondrial fission and subsequent mitochondrial autophagy. After infection, HCV promotes the activity of the CDK1/cyclin B complex, induces the phosphorylation of Drp1Ser616, and stimulates the expression of Drp1 and its receptor protein Mff, both of which enhance the migration of Drp1 to mitochondria, leading to enhanced mitochondrial division (Kim et al., 2014). In addition, HCV was also shown to cause mitochondrial dysfunction such as membrane potential loss (Hino et al., 2019) and oxidative phosphorylation dysfunction. These damaged mitochondria may also be the cause of mitochondrial fission. Subsequently, the virus up-regulated the expression of PINK1 and Parkin genes and the mitochondrial recruitment of Parkin, which triggered the occurrence of mitochondrial autophagy (Seong-Jun et al., 2013; Kim et al., 2014), cleared the damaged mitochondria, and prevented the release of pro-apoptotic proteins, thus inhibiting cell apoptosis and ensuring the long-term survival of the virus. When Drp1 was silenced, HCV-induced mitochondrial autophagy was significantly inhibited, the cell showed apoptotic signals, and the secretion of virions was reduced, which indicated that mitochondrial fission was an early event of mitochondrial autophagy and had important significance in the persistent infection of HCV. Similar results were seen in cells that were infected with hepatitis B virus (HBV) and porcine reproductive and respiratory syndrome virus (PRRSV; Shuaifeng et al., 2016; Kim et al., 2017).

In HCV-related studies, many viral proteins were found to play important roles in inducing mitochondrial fission and autophagy. For example, envelope glycoprotein 2 (E2), core protein, and non-structural protein 3/4A (NS3/4A) can all induce phosphorylation of Drp1S616 (Kim et al., 2014), and non-structural protein 5A (NS5A) leads to loss of mitochondrial membrane potential (Jassey et al., 2019). Further investigation of the function and the exact mechanism of these viral proteins, as well as substances that can inhibit their activity, may provide new ideas for the treatment of chronic hepatitis and drug research and development.

In the later stage of infection, viruses release virus particles by inducing apoptosis. Rotavirus (RV) is a typical case of promoting apoptosis in the late stage of infection. Mitochondrial fission was shown to be a prerequisite for RV to induce apoptosis, and the level of Drp1 has a significant regulatory effect on apoptosis (Arpita et al., 2018). In the late stage of RV infection, viral nonstructural protein 4 (NSP4) induces phosphorylation of Drp1S616 through CDK1, and NSP4 is transferred to mitochondria to participate in recruitment of Drp1 and increase splitting activity. Subsequently, mitochondrial translocation of E3 ubiquitin ligase Parkin was observed, targeting Mfn1 to degrade it by ubiquitination, further promoting mitochondrial fission (Rahul et al., 2012; Arpita et al., 2018). Next, the Cyt C is released from the fragmented mitochondria into the cytoplasm, and the activity of caspase-3 and caspase-9 increases, thereby initiating the apoptosis cascade reaction and spreading mature virus particles to surrounding cells to promote transmission of the virus offspring (Arpita et al., 2018). Many studies have found that excessive mitochondrion division can lead to apoptosis, and it is a crucial process in apoptosis. In the early days, it was thought that mitochondrial fragmentation mediated by Drp1 would lead to the release of CytC and other apoptosis factors (Frank et al., 2001), but subsequent studies found that inhibiting mitochondrial division only reduced the release of CytC but had no effect on the release of other apoptosis factors in mitochondria, which indicated that mitochondrial division may not be the main cause of CytC release (Parone et al., 2006); there is also a view that mitochondrial division will play an important role only when a large amount of CytC is needed to activate caspase (Martinou and Youle, 2006). Other studies have found that the degradation of the fusion protein L-OPA1 will lead to the damage of mitochondrial cristae, thus triggering the release of CytC. Therefore, the relationship between mitochondrial division and CytC release and its role in the occurrence of apoptosis need to be further explored.

Above, mitochondrial fission has a different regulatory effect on apoptosis in different infection periods, which may be the result of the interaction of viral factors and intracellular signaling molecules, and it is also related to different apoptotic signaling pathways. With research advancements, the relationship between viral infection and apoptosis and the role of mitochondrial division are gradually being clarified. Researchers have applied viral products that promote apoptosis or inhibit apoptosis at different stages of viral infection to treat the infection based on the mechanism of action of the virus studied. Future research can take this as a direction to further study the relationship between viral infection and apoptosis, as well as the interaction between viral products and intracellular molecules to develop new antiviral agents.

### Viruses Disturb Mitochondrial Dynamics to Inhibit RLR Antiviral Signaling Pathway

**Figure 3:**
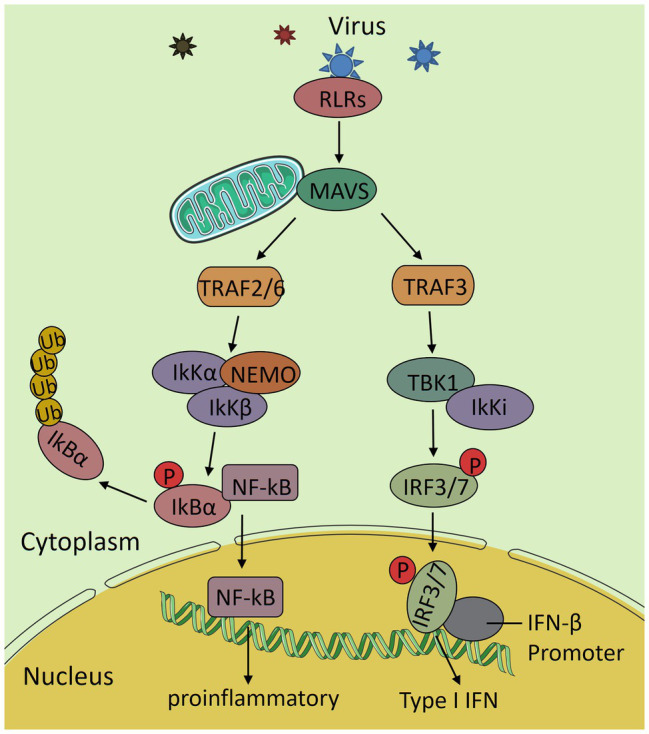
The RIG-I-like receptors (RLRs) antiviral signaling pathway. After the virus invades the body, RLRs recognizes the viral RNA and interacts with mitochondrial antiviral signaling (MAVS) to activate it. Then, it transduces the signal to the downstream TNF receptor associated factor 2/6 (TRAF2/6) and TRAF3, further activates IκB kinases (IkK complex) and TANK-binding kinases (TBK1) complex and nuclear transcription factor κB (NF-κB) and interferon regulatory factor 3 (IRF3), and induces the expression of various proinflammatory factors and interferon and finally produces an efficient antiviral immune response.

When the body is infected by viruses, RIG-I-like receptors (RLRs) recognize the viral RNA in the cytosol and undergo conformational changes to interact with the caspase recruitment domains (CARD) of the MAVS protein, which recruits TANK-binding kinases (TBK1) and IκB kinases (Iκk) to form complexes that activate interferon regulatory factor 3 (IRF3) and nuclear transcription factor κB (NF-κB), respectively. This signaling further initiates the downstream interferon (IFN) response and inflammatory response to increase the expression of INF and various proinflammatory factors, which results in a highly effective antiviral immune response (Loo and Gale, 2011; Figure 3). In recent years, it has been found that the prohibitin complex on mitochondria can bind to MAVS through the bridging of caseinolytic peptidase B protein homolog (CLPB) and act as a signal body to recruit downstream proteins (Yoshinaka et al., 2019). The RLR signaling pathway is part of innate immunity in the body, which is the first line of defense in the immune system. When this pathway is destroyed, it inhibits the expression of interferons and pro-inflammatory factors and finally leads to a decrease in immunity in the body. There is an interaction between the RLR signaling pathway and mitochondrial dynamics. In most cases, the extension of the mitochondrial network enhances the transmission of antiviral signals, while mitochondrial fragmentation has the opposite effect, and activation of RLR pathway also promotes mitochondrial fusion (Castanier et al., 2010).

Cytomegalovirus (CMV) is a member of the herpes virus family, which can infect many animals and cause serious damage to many systems in the body. CMV has been shown to induce mitochondrion fission and block the antiviral signaling pathway by targeting MAVS and stimulator of interferon genes (STING; Castanier et al., 2010). STING is an important natural immune signal molecule, which is located on mitochondrial-associated membrane (MAM) that is in contact with ER, which forms a complex with MAVS and Mfn1. When the RLR pathway is activated, the complex degrades and initiates MAVS-STING interaction, as follows: STING recruits TBK1 as a linker protein and connects it to MAVS, thus activating IFN response (Ishikawa and Barber, 2008; Brubaker et al., 2014). After CMV infection, the viral protein vMIA triggers mitochondrial fission, reduces the contact between ER and mitochondria, and inhibits the interaction between MAVS and STING and, thus, inhibits the downstream signaling of MAVS. It also delays the degradation of the MAVS complex, inhibits the release of Mfn1, and further aggravates mitochondrial fragmentation (Castanier et al., 2010). After infection with influenza A virus (IAV), the viral protein PB1-F2 transfers to the IMM *via* the translocase of the outer mitochondrial membrane 40 (TOM40), activates OMA1 located in the inner membrane, and then shears L-OPA1, resulting in fragmentation of mitochondria (Yoshizumi et al., 2004); PB1-F2 has also been proved to bind to MAVS, thus inhibiting RLR-dependent antiviral immune response (Varga et al., 2012), but it is still unclear whether there is a functional relationship between mitochondrial fragmentation mediated by PB1-F2 and innate immune impairment.

It has been found that some viruses can upregulate intracellular microRNA (miRNA) after infection so as to regulate the process of virus infection. miRNA is a non-coding RNA with a length of about 20–25 nucleotides, which influences various cell activities by regulating the expression of genes in eukaryotic cells. When Sendai virus (SeV) was used to attack cells, the expression of various miRNA was upregulated, among which miR-302b and miR-372 both inhibited the expression of phosphorylated Drp1 at S637 site and caused abnormal mitochondrion fission, thus inhibiting part of RLR signaling (Yasukawa et al., 2020). Subsequently, it was found that these two miRNAs also downregulated some mitochondrial metabolism-related proteins and a solute carrier protein SCL-25 A-12, which can act on MAVS downstream, further destroying the antiviral immune response of mitochondria (Yasukawa et al., 2020). Another miRNA (Mir-3470b) was upregulated after infection with bovine ephemeral fever virus (BEFV) and promoted viral replication by targeting MAVS to inhibit antiviral signal transduction (Hou et al., 2018). In recent years, the regulation of miRNA has become a research hotspot, and literature has reported the induction or protection of miRNA during the occurrence of various diseases (Du et al., 2019; Kumar et al., 2019), which suggests that different miRNAs may be the key targets for future treatment of various diseases.

Although mitochondrial elongation is generally believed to promote MAVS-mediated antiviral immune responses, mitochondrial fusion can also disrupt this innate immunity in the context of certain viral infections. Dengue virus (DENV) is an RNA virus. When DENV invades cells, it uses ER-derived structures in host cells to translate various viral proteins for assembly of its own viral particles or replication of viral genes (Chatel-Chaix et al., 2016). Convoluted membranes (CMs) are a substructure that is derived from the ER, and they play an important role in the maturation of DENV proteins (Welsch et al., 2009). The formation of CMs also destroys the contact interface between the ER and the mitochondria on MAM, thereby inhibiting the shift of RIG-I to MAVS on MAM and weakening the signal transmission of MAVS (Chatel-Chaix et al., 2016). Laurent et al. found that after DENV infection, by reducing the phosphorylation level of Drp1S616, the mitochondrial elongation caused by DENV can promote the formation of CMs, thus inhibiting the antiviral immunity of the body (Chatel-Chaix et al., 2016; Barbier et al., 2017). However, in another study on DENV, mitochondrial fragmentation was observed after infection, which was shown to be caused by viral protein NS2B3, and this cuts Mfns in host cells (Yu et al., 2015). This reminds us that different strains or cell lines may produce different experimental results and further experiments are needed to determine the specific reasons for this difference.

### Viruses Disturb Mitochondrial Dynamics and Causes Neuronal Damage

Neurons are the most basic structural and functional units in the nervous system with the function of receiving, integrating, transmitting, and exporting information to achieve information exchange. Mitochondria are the energy-supplying organelles in neurons and are transported *via* the microtubule structure to the dendrites and axons that have the highest energy demand to ensure the normal function of neurons (Chang et al., 2006). The study found that abnormal mitochondrial fission and fusion can lead to the disruption of mitochondrial transport and impaired respiratory function, leading to the injury or death of neurons, which is also the cause of many neurodegenerative diseases.

Human immunodeficiency virus (HIV) invades the central nervous system of the body after infection, causing neuronal damage and eventually a series of neurological syndromes (Spudich and González-Scarano, 2012). After HIV infects cortical neurons in rats, the viral protein gp120 downregulates the expression of Fis1, and transactivator of transcription (TAT) inhibits phosphorylation of Drp1 S637 residues, synergistically destroying the balance of mitochondrion division and fusion, thereby causing different degrees of functional and structural damage (Avdoshina et al., 2016; Rozzi et al., 2018). These damaged mitochondria lose motility, stop bidirectional transportation, and gather in the cell body, and the axon and dendrite lose their energy supply and begin retrograde degeneration, eventually causing neuron damage and death (Zheng et al., 2004). In addition, mitochondria produce a large amount of ROS because of respiratory dysfunction, which further promotes the nerve cell death (Wu et al., 2005). Similar findings have been found in the rodent superior cervical ganglion (SCG) that is infected with the pseudorabies virus (PRV). Mitochondrial transport was inhibited after dynamics imbalance, and only 5% of the mitochondria retained their motility, which is necessary for PRV growth and transmission (Kramer and Enquist, 2012).

Abnormalities in mitochondrial dynamics have been observed in many neurodegenerative diseases, such as Alzheimer’s disease (AD) and Parkinson’s disease (PD; Su et al., 2010; Sandra, 2011), suggesting that the balance of mitochondrial dynamics in neurons is important. Therefore, research on such diseases may start from the mitochondria and continue with the study of the molecular regulatory mechanisms of mitochondrial damage. This will help to improve our understanding of the pathogenesis of neurological diseases.

### Viruses Disturb Mitochondrial Dynamics to Promote Cell Migration

Cell migration is the movement of cells after receiving certain migration signals. It is a common form of cell movement and is closely related to a variety of physiological activities. Malignant migration of cells is the main cause of cancer death, and some studies have found that mitochondrial dynamics and peripheral distribution will affect cell migration (Zhao et al., 2013; Yasuhito et al., 2018). In PRCC-TFE3 translocated renal cell carcinoma and metastatic breast cancer cells, Drp1-mediated mitochondrion division increases, thus promoting the migration and invasion of cancer cells (Zhao et al., 2013; Bo et al., 2020).

Epstein-Barr virus (EBV) is a carcinogenic virus that is associated with a variety of epithelial and lymphoid malignancies. Its latent membrane protein upregulates Drp1 expression through the Notch pathway. Additionally, the increase in Drp1 expression leads to an increase in cell migration and epithelial–mesenchymal transformation, thus increasing the invasion force of the virus (Deb et al., 2014). Another study found that fragmented mitochondria led to an increase in cell lamellar lipid layer formation (Zhao et al., 2013), which is an important structure for cancer cell migration and invasion. The lipid layer usually adheres to the substrate and generates the driving force to move cells forward (Hideki and John, 2007), which may explain why mitochondrial division promotes cell migration. However, there is no definite research to show that mitochondrial fission caused by EBV can produce this effect, and more evidence is needed to confirm this hypothesis.

### Viruses Disturb Mitochondrial Dynamics to Promote Cell Syncytium Formation

Syncytium is a multinucleated giant cell that is produced by the fusion of two or more cells under natural conditions or artificial induction. The formation of syncytium causes pathological changes in normal cells or tissues. Some viruses, which are called syncytial viruses, can cause this cell fusion.

Newcastle disease virus (NDV) is a syncytial virus, and its fusion protein (F) and hemagglutinin-neuraminidase (HN) induce syncytium formation, thus causing a series of cytopathic effects (CPE) and cell death (Ravindra et al., 2009). Ren et al. (2019) found that, in this process, the synergistic effect of F and HN upregulated the expression of a variety of dynamic proteins and changed the distribution and morphological characteristics of mitochondria by disturbing their dynamic balance to meet the energy requirements for membrane fusion to promote and assist with syncytia formation. Some syncytial viruses can remove diseased tissues or tumors (Schirrmacher and Fournier, 2009). Whether the cell fusion caused by these viruses can be regulated by mitochondrial dynamics and further applied to tumor biotherapy requires more *in vivo* and *in vitro* studies.

### Viruses Disturb Mitochondrial Dynamics to Promote Cell Release of Extracellular Microvesicles

Extracellular microvesicles (EMVs) are small vesicles that fall off the cell membrane after activation, injury, or apoptosis. They can encapsulate proteins, lipids, nucleic acids, and other substances and transport them to other cells through release to mediate information transmission between cells. Many envelope viruses take advantage of EMVs to pack virus proteins and genomes into them and release them from cells, thus promoting viral spread and persistent viral infection (Izquierdo-Useros et al., 2009; Ariumi et al., 2011).

It is generally believed that non-envelope viruses release virus particles through cell lysis. However, it has been found that several non-envelope viruses can release EMVs by hijacking the host cell membrane structure (Scott et al., 2014; Chen et al., 2015). Among them, the process of EMV release by coxsackievirus B (CVB) is closely related to mitochondrial dynamics (Jon et al., 2017). Mitochondrial fragmentation is triggered by CVB, which is effectively enhanced by mitochondrial autophagy, and the virus uses the autosomal membrane structure as its own EMV raw material to package and release virions. Further research found that after silencing *Drp1* expression, CBV transmission was well controlled (Jon et al., 2017). This study provides a way for non-envelope viruses to release EMVs and shows its functional relevance to mitochondrial dynamics. The results may be applicable to other non-envelope viruses and provide new strategies for their treatment.

## Conclusions and Perspectives

After viral infection, there is a series of physiological changes in host cells. These changes include viral-induced changes to the mitochondria and destruction of the mitochondrial dynamic balance, which benefit the virus. Based on the literature that we have reviewed, viruses mainly interfere with mitochondrial dynamics in the following ways: (1) post-translational modification of dynamic proteins, (2) regulation of dynamic protein expression, (3) cleavage of mitochondrial dynamic proteins, and (4) changes in the physiological environment within cells to interfere with mitochondrial dynamics. Through the above effects, the virus indirectly regulates cell apoptosis, inhibits the RLR antiviral signaling pathway, promotes tumor cell migration or promotes syncytium formation to upregulate viral infection processes and cause damage to the host’s body. In recent years, several studies have reported the association of cGAS-STING pathway between mitochondrial homeostasis and antiviral activity: after excessive mitochondrion fission or inhibition of autophagy leads to a large accumulation of damaged mitochondria, mtDNA in cytoplasm increases abnormally and is recognized by a DNA receptor cyclic GMP-AMP synthase (cGAS), and then cGAS catalyzes the synthesis of cyclic GMP-AMP (cGAMP), which further activates STING, then STING recruited and activated TBK1, thus inducing interferon production (Newman and Shadel, 2018; Yang et al., 2018). However, the above effects were found in the context of disease occurrence. It is still unknown whether mitochondrial dynamics can be regulated to enhance the antiviral ability of the body through the Cgas-STING pathway under physiological conditions. In the future, we can further study the mechanism of mtDNA increase in cytoplasm caused by mitochondrial damage, which will help us better understand the relationship between mitochondrial dynamics and cGAS-STING pathway.

Although much progress has been made in this field, there are still many unsolved problems. These unsolved problems include few studies that mention the effects of the mitochondrial dynamic balance that is manipulated by viruses on the mitochondria and cellular physiological functions and the mechanism of interaction between mitochondrial dynamic proteins and viral factors. Understanding these problems may improve our understanding of viral pathogenesis. In future studies, abnormal changes in mitochondrial dynamic proteins may be used as an indicator to predict damage to the body after viral infection, and the corresponding treatment may be given. In addition, the functional correlation between mitochondrial dynamics and viral pathogenesis should be further elucidated to develop new antiviral treatment strategies and new and efficient antiviral drugs.

## Author Contributions

ZR: conceptualization, writing-review and editing, and revised the manuscript. XZ: writing-original draft and revised the manuscript. TD: writing-review. ZZ: software. HH: validation. ZX, JD: supervision. All authors contributed to the article and approved the submitted version.

### Conflict of Interest

The authors declare that the research was conducted in the absence of any commercial or financial relationships that could be construed as a potential conflict of interest.
